# PaSS: a sequencing simulator for PacBio sequencing

**DOI:** 10.1186/s12859-019-2901-7

**Published:** 2019-06-21

**Authors:** Wenmin Zhang, Ben Jia, Chaochun Wei

**Affiliations:** 1 Department of Bioinformatics and Biostatistics, School of Life Sciences and Biotechnology, Shanghai Jiao Tong University, Shanghai, 200240 China; 2 Shanghai Center for Systems Biomedicine, Shanghai Jiao Tong University, 800 Dongchuan Road, Shanghai, 200240 China

**Keywords:** Third generation sequencing, Next generation sequencing, PacBio sequencing, Sequencing simulator, Sequencing error, Sequence pattern

## Abstract

**Background:**

Third-generation sequencing platforms, such as PacBio sequencing, have been developed rapidly in recent years. PacBio sequencing generates much longer reads than the second-generation sequencing (or the next generation sequencing, NGS) technologies and it has unique sequencing error patterns. An effective read simulator is essential to evaluate and promote the development of new bioinformatics tools for PacBio sequencing data analysis.

**Results:**

We developed a new PacBio Sequencing Simulator (PaSS). It can learn sequence patterns from PacBio sequencing data currently available. In addition to the distribution of read lengths and error rates, we included a context-specific sequencing error model. Compared to existing PacBio sequencing simulators such as PBSIM, LongISLND and NPBSS, PaSS performed better in many aspects. Assembly tests also suggest that reads simulated by PaSS are the most similar to experimental sequencing data.

**Conclusion:**

PaSS is an effective sequence simulator for PacBio sequencing. It will facilitate the evaluation and development of new analysis tools for the third-generation sequencing data.

**Electronic supplementary material:**

The online version of this article (10.1186/s12859-019-2901-7) contains supplementary material, which is available to authorized users.

## Background 

Third-generation sequencing technologies including the PacBio or SMRT (single-molecule real-time) sequencing and nanopore sequencing are causing a revolution in genomics study as they provide researchers to study genomes at an unprecedented sequencing read length [1]. SMRT sequencing developed by Pacific BioSciences is among the most widely used third-generation sequencing technologies [2].

More and more bioinformatics tools and algorithms, such as sequence alignment program BLASR [3] and GraphMap [4], genome assembly program canu [5] and miniasm [6] and structural variant callers PBHoney [7] and Sniffles [8] have been emerging for SMRT data analysis. Besides, PacBio sequencing has been developed quickly with multiple versions. It’s essential that these tools are benchmarked and assessed using reads simulated by sequencing simulators targeting on a specific version of PacBio technology. The simulation of PacBio data can be useful to guide users to choose the most appropriate analytical tool or approach for their own research projects [9]. In addition, generating in silico data can significantly reduce the cost and time required for improving the downstream analysis tools [10].

The characteristics of PacBio reads is quite different from that of the second-generation sequencing reads’. It is capable of producing reads about 10-15 kb, which is much longer than existing second-generation sequencing methods’. Long reads can be useful for spanning repetitive or complex regions such as large structural variations since the mapping position of a read in a genome can be determined more precisely. Therefore, long reads show superiority in the analysis of repetitive regions and large structural variations. In contrast, it is difficult for the second-generation sequencing that may lead to misassembles and gaps. However, the per-base error rate can be about 15% compared to less than 1% in the second-generation sequencing technology and the errors are dominated by indels [11]. Nevertheless, the high error rate can be alleviated by the single-molecule circular sequencing or multi-pass sequencing. In the sequencing process, the forward and reverse strands of the target molecule can be sequenced multiple times using the circular template [12]. The output sequence termed as a polymerase read can be split into multiple reads called subreads. The final output sequencing read quality can be improved by generating consensus of these subreads. Although the throughput is still low, the latest sequencer Sequel can generate seven-fold to ten-fold more sequences than the older sequencer PacBio RSII. It can produce 5-10Gb bases with about 365 k~ 500 k reads per run [13]. Furthermore, compared to the NGS methods, PacBio sequencing is faster and has no GC bias [14].

Currently, there are several tools to simulate PacBio reads, such as PBSIM [15], LongISLND [16], and NPBSS [17]. All these simulators can estimate the read length distribution but only the LongISLND considers multi-pass sequencing of PacBio platform. PBSIM can simulate reads using either a model-based or sampling-based method. But the read length distribution of PBSIM does not match current data well. LongISLND employs a sequence context sensitive method called extended-kmer to deal with the homopolymer-dependent bias and it can output in multiple file formats. NPBSS can use the relationship between the real error rate and quality values (QVs) while it takes a long time in simulating. For the sequences from the latest sequencer Sequel, a fixed quality value (QV) was used so the QVs do not represent the actual error rates whereas the methods of PBSIM and NPBSS simulating sequencing errors are based on QVs. In addition, LongISLND cannot process the file format of Sequel data. Most of all, these three simulators built their sequencing error models based only on the aligned regions from alignment results, thus some information about the sequencing error, especially those regions with low qualities, were missing.

In order to catch the innovation of sequencing technology and improve existing methods, we propose a new PacBio sequence simulator PaSS. PaSS can generate customized sequencing pattern models from real PacBio data and use a sequencing model, either customized or empirical, to generate subreads for an input reference genome. Finally, PaSS and some popular existing simulators are compared. The results and the assembly tests show that PaSS can simulate PacBio reads with high fidelity.

## Implementation

In general, PaSS can produce in silico reads using sequencing error models built previously for some given reference genomes. Sequencing error models can also be re-estimated from a real PacBio sequencing data. The methods involved are introduced as follows.

### Estimating sequencing error models from real sequencing data

In order to simulate PacBio sequencing better, the multi-pass sequencing of PacBio sequencer was investigated. We noticed that there is a trade off between target read length and the number of passes that longer template will be cycled less. PaSS can learn the read sequence patterns from real sequencing data. The distributions of pass-numbers and their corresponding read length distributions are recorded in the model for sequence generation.

**Fig. 1:**
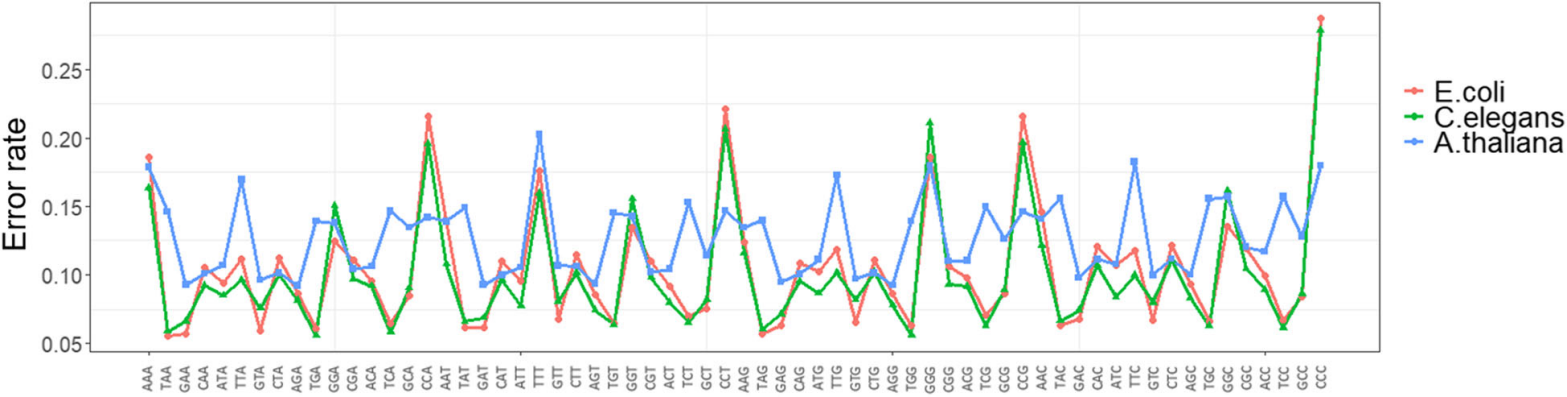
The distribution of sequencing error rates based on the context of 64 k-mers (k = 3) in real sequencing datasets of *E. coli K12*, *C. elegans* and *A. thaliana*

In order to learn how the errors were distributed across a read, we aligned PacBio reads to the reference sequences. After we tried multiple alignment tools for long reads, we adopted BLASR [3] to align sequencing reads to a reference genome or a high-quality de-novo assembly. The alignment results of real sequencing data are analyzed to extract sequencing error models which can be served as the input at the simulation stage. The head and tail regions of some reads may not be aligned back to the reference sequences because of the high error rates on these regions. A ratio of the unaligned part of a whole polymerase read is estimated to get a more integral model (see Additional file 1: Figure S1). The average sequencing quality over the whole read varies from reads to reads. PaSS learns the ratio of different error types (match/insertion/deletion/substitution), and their corresponding sequence context patterns by kmer-based analysis. Table S1 shows the 64 k-mers (k = 3) frequencies in different error events for real sequencing data. Every event is recorded with its corresponding 3-base sequence in reference and the continuous error is regarded as one event. Error rates are relatively high on some k-mers especially those k-mers whose first two bases are the same. The tendencies (Fig. 1 and Additional file 1: Figure S2) in datasets *E. coli* and *C. elegans* seem close and it’s reasonable because these two datasets are from the same sequencer RSII P6-C4. The error size distribution is also derived from alignment results. Although we observed sequencing error bias across the relative locations in the reads, we did not include this pattern in the current version of PaSS.

### Simulation of PacBio multi-pass sequencing reads

**Fig. 2:**
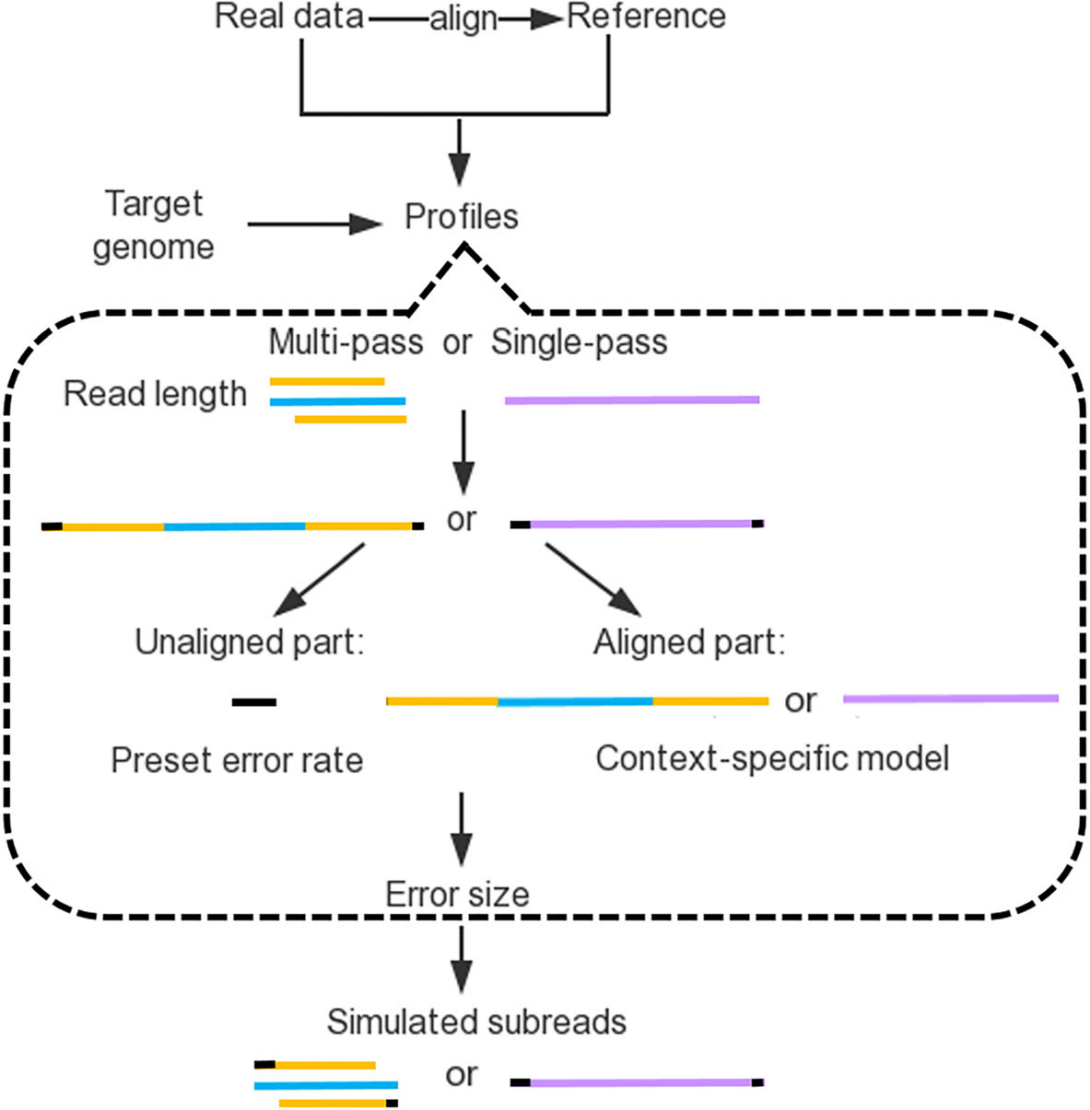
System diagram of PaSS. The sequencing profile (or sequencing error model) can be generated from real sequencing data and  its alignment to a reference genome. Reads can be simulated based on the reference genome and the sequencing profile (or error model). For each read, a fragment of sequence from the reference genome is selected first then the sequencing errors will be added according to the profile, which includes preset error rates for two ends of the read, the number of passes, read length distribution, context based error model and error size

Figure 2 illustrates the simulation process. First, the number of forward-reverse cycles is estimated from the distribution of pass-number and the read length is determined by the corresponding length distribution of this pass-number. PaSS then randomly samples one error-free read from a user-specified reference genomic sequence. If the selected sequence contains Ns, those Ns are replaced randomly with ACGTs in the read. The collected read is treated as a sequence template, and the subreads of it alternate between the forward and reverse strands. Finally, errors are introduced to get the output read. The reads that are marked to come from the same template are divided into presumed unaligned part and aligned part according to the relative position inside the polymerase read. For the presumed unaligned sections, we use a preset high error rate. According to the comparison between different preset error rates (Additional file 1: Table S2), we chose 0.4 as the default value. As for the aligned regions, an event type is randomly drawn based on the context-specific bin recorded in the model. When an error occurs, the length of the error is then drawn from the model. From the real PacBio data, we found that the inserted nucleotides depend on the sequence context. Hence, if the error is an insertion, the inserted nucleotides are also decided by the context. If the error is a substitution, the substitution pattern is introduced according to the distribution of twelve substitution types.

### Real PacBio sequencing datasets

In order to assess the performance of PaSS, three real PacBio sequencing datasets for *E. coli*, *C. elegans*, and *A. thaliana* were chosen for benchmarking. Additional file 1: Table S3 shows brief statistics of these datasets and they can be downloaded freely from the websites listed in Additional file 1: Table S3. In order to have a comprehensive assessment of the performance of sequencing simulators, we included real sequencing data from two different platforms, RSII and Sequel. The sequencing data for *E. coli* and *C. elegans* were from RSII sequencing platform while *A. thaliana* sequencing data was from the latest Sequel platform.

### Simulation method comparison

In order to do a fair comparison, we tried to estimate the sequencing models for all methods from the real sequencing data first and reads were simulated using the sequencing models generated for the same genome. Since the NPBSS program can only simulate single chromosome, we simulated a chromosome each time and mixed the reads for *C.elegans* and *A. thaliana*. LongISLND couldn’t generate profile from Sequel data and we did not simulate reads for *A. thaliana* using LongISLND.

## Results and discussion

A new sequencing simulator for PacBio sequencing called PaSS was implemented. We compared PaSS with three existing popular methods, PBSIM, LongISLND and NPBSS, using three sequence datasets (see Methods for more details).

### Simulation results and comparisons

**Fig. 3:**
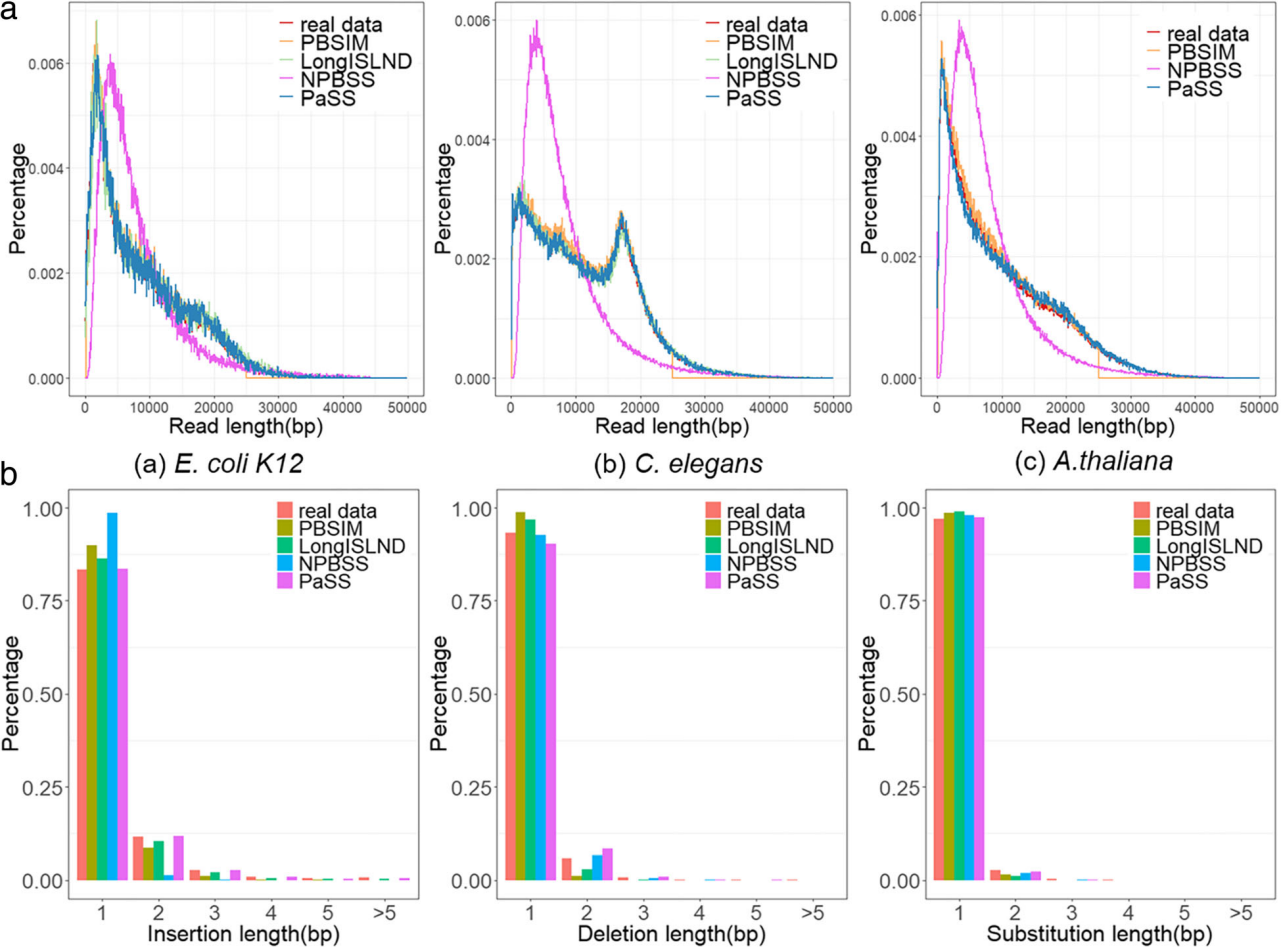
Comparison of simulated data with the real sequencing data. (**a**) Comparison of read length distributions. Subfigure a, b and c showed the read length distribution collected for three organisms, *E. coli* *K12*, *C. elegans* and *A. thaliana* respectively. (**b**) Comparison of error size distribution. Simulated reads from different methods were compared with real sequencing data on error size distribution for organism *E. coli* *K12*. Three subfigures showed the probability density bar plot for insertion, deletion and substitution respectively

The length distribution of simulated reads and that of the real sequencing data were compared and results were shown in Fig. 3 (A). All simulators get length distribution similar to that of real sequencing data. The default value of maximum read length defined in PBSIM was outdated and could not be reconfigured.

We then assessed the length distribution of the error bases. Figure 3 (B) shows the length distribution of the error bases for *E. coli* (Additional file 1: Figure S3. for *C. elegans* and *A. thaliana*). Although most lengths of indels are one base long, there are about 15–20% of insertions and 7–10% of deletions contain multiple bases. Unlike the indels, the length for vast majority of the substitutions is one base. PBSIM and NPBSS reads are more distinct from the real sequencing data at this aspect because they only include single base error in their models.

Next, we used the Kolmogorov–Smirnov test (K-S test) to decide whether two probability distributions of real sequencing data and simulated data are different. The null hypothesis of this test is that the two sets of data are drawn from the same distribution. K-S test was performed for the read length distribution and the distribution of error bases. The resulted *p*-values in Table S4 and S5 (see Additional file 1) reject the null hypothesis, which indicate the two distributions between all the simulated data and real sequencing data are distinguishable. Nevertheless, the test statistics D between real sequencing data and simulated data of PaSS is the minimum among the several simulators in most cases. Test statistics D is the maximum value of the difference between two distributions. Therefore, it shows that the distance between the distributions of simulated data from PaSS and real data is the closest. Moreover, it is consistent with what is shown in Additional file 1: Figure S4 and S5.

Table 1 (Additional file 1: Tables S6-S7) shows the statistics of alignment results, from which we can see that the alignment rates and the error rates in terms of insertion, deletion and substitution from PaSS are more consistent with the real sequencing data than existing methods. More than 99% bases of the simulated reads by PBSIM, LongISLND and NPBSS can be aligned to the reference, while the alignment rates of real sequencing reads and PaSS reads are more consistent to each other, ranging from 89 to 94% for the three datasets. Because only the aligned regions were analyzed and included in the estimated profile, the unaligned regionswere ignored by these three simulators. As mentioned before, the quality values (QVs) in Sequel sequenced data did not represent the actual error rates. Therefore, PBSIM was not able to get reasonable parameters from real sequencing data from the Sequel platform. If we did use PBSIM to re-estimate sequencing error models from real Sequel sequencing data, the error rate may be less than 1%, which was much lower than it should be. Additional file 1: Figure S6 showed the distribution of the average accuracy (1-error rate) over the whole polymerase read. The quality of sequencing reads is not uniform and PaSS provides a more realistic simulation result than other tools. In general, PaSS can simulate PacBio data reasonably better than other simulators especially for the new Sequel data.

**Table 1:** Statistics about the simulated reads by PBSIM, LongISLND, NPBSS, PaSS and real sequencing data for *E. coli K12* genome. Reads were aligned back to *E. coli* *K12* genome

methods	aligned rate(read)	aligned rate(base)	error rate	insertion	deletion	substitution
real data	96.71%	91.74%	14.54%	9.42%	3.86%	1.27%
PBSIM	99.99%	99.73%	12.27%	7.22%	3.16%	1.89%
LongISLND	99.90%	99.92%	11.07%	7.09%	2.77%	1.20%
NPBSS	100.00%	99.93%	11.48%	2.67%	6.05%	2.76%
PaSS	95.84%	92.53%	14.39%	8.97%	3.80%	1.62%

We further investigated the correlation between the error rates and the relative positions of bases in a read. We divided each polymerase read into ten fragments equally, then calculated the average error rate of each fragment. As shown in Additional file 1: Figure S7, the error rate from the real sequencing data decreases quickly at the first one or two fragments and then increases slightly at the end fragment of a read. We have tried to divide the reads into 10 evenly divided intervals, and one interval, and found that the simulation results for the 10 interval model and the one-interval model were similar. Therefore, we adopted one interval model in the end.

### Speed comparison

In order to compare the speed of the four simulators, we simulated reads for genome *E. coli K12* and *C. elegans* with sequencing depth 170 and 50 respectively. We report the computational time for all simulators in Additional file 1: Table S8. PaSS can be run in parallel with multi-thread. Therefore, different running time for PaSS was listed with various numbers of threads. PBSIM is the fastest tool while NPBSS is the slowest one. PaSS is faster than PBSIM and LongILSND if more than 4 threads are used for PaSS.

### Assessment of simulation with assembly results

**Fig. 4:**
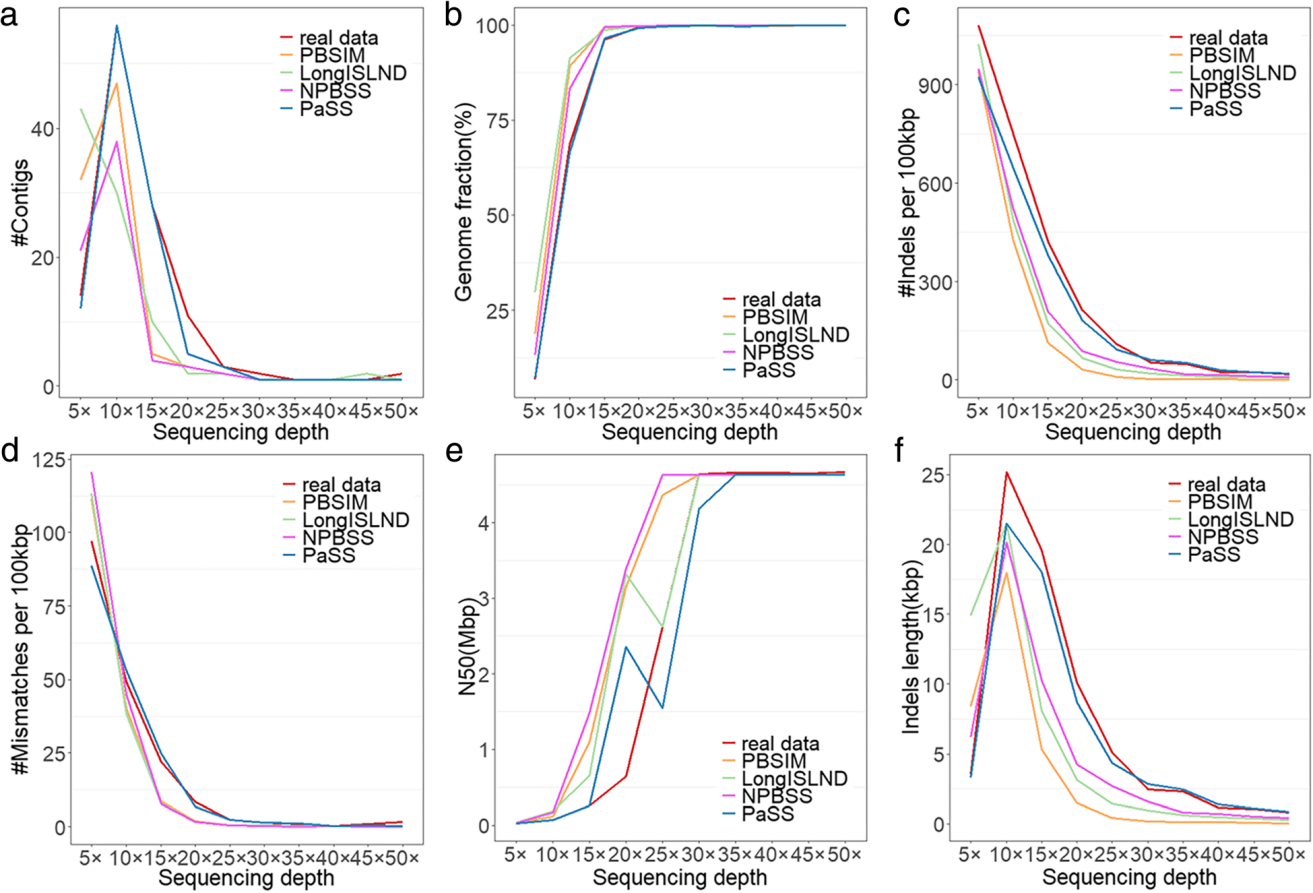
Comparison of the assembly results for real reads and simulated by different methods. The six subfigures show the relationships between sequencing depth and the resulted (**a**) number of contigs, (**b**) genome fraction, (**c**) number of indels per 100kbp, (**d**) number of mismatches per 100kbp, (**e**) N50, and (**f**) the length of indels of contigs in the assembly results for organism *E. coli K12* . X-axis stands for the sequencing depth

We conducted assembly on reads simulated by PBSIM, NPBSS, LongISLND and PaSS and real sequencing data. Each genome was simulated with sequencing depths of 5, 10, 15, 20, 25, 30, 35, and 40. *C. elegans* was simulated additionally for sequencing depth of 45 and *E. coli* was simulated additionally for sequencing depth 45 and 50. We conducted de novo assemblies using canu, an assembler designed for noisy long length read sequencers [18]. Quast [19] was utilized to compare the assemblies with the reference genome and evaluate the simulators in terms of some features. The number of contigs, genome fraction, indels per 100 kb, mismatches per 100kbp, N50 and indel length of the contigs were compared for assemblies derived from simulated datasets and the real sequencing data. The assembly results shown in Fig. 4, Additional file 1: Figure S8 and S9 are for *E. coli K12*, *C. elegans* and *A. thaliana* respectively. Assembly result evaluation is an indirect comparison between the simulators. The results of PaSS show more similar patterns to the results of real sequencing data than other simulators. In terms of the number of contigs in the assembly and genome fraction being assembled, the curve tends to be stable at 25× for organism * E. coli* and 35× for organism *C. elegans*. It indicates that these sequencing depths can be adopted in real experiments. From the proportion that the reference covered by the number of aligned contigs, the assembly result of PaSS is much closer to real sequencing data than the other simulators. What’s more, the indicators about the average number of indels and mismatches per 100kbp aligned bases also show that PaSS simulated reads are more similar to the real data than other simulators. Although the contigs assembled from high sequencing depth (>30X) simulated data could cover most of the reference genomes for all simulators, the results from lower sequencing depth (5-30X) did show the gap between the real data and simulated data, and the simulated reads from PaSS were more similar to the real data than the other simulators.

## Conclusions

In this paper, we propose PaSS to simulate PacBio sequencing reads to keep up with the latest sequencing technology. We incorporated sequence context into the sequencing model of PaSS. Comparing to existing methods, the part of the sequence that cannot be aligned back to reference due to the high error rate is also considered in PaSS. To our evaluation, PaSS can simulate PacBio sequencing reads more similar to real PacBio data than the existing simulation systems. Overall, PaSS is an effective sequence simulator to generate benchmark datasets with the known ground truth so that it can be beneficial to evaluate the latest bioinformatics tools. Besides, it can be used as guidance for researchers since no gold standard is available for data analysis. The simulation of PaSS could serve as a reference to determine some critical parameters for a specific project.

However, PaSS can still be improved on various aspects. First,the length of kmer is limited. Second, the method to estimate error models based on alignment results is not perfect. The algorithm and performance of the alignment tool will affect the estimated error models and may bring additional bias. Third, the sequencing simulator cannot be customized or updated easily for different species.

## Availability and requirements

**Project name:** PaSS.

**Project home page:** http://cgm.sjtu.edu.cn/PaSS

**Operating system(s):** Linux.

**Programming language:** Perl and C.

**Other requirements:** Perl(5.10.1 or above), gcc (4.8.0 or above).

**License:** GNU GPL.

**Any restrictions to use by non-academics:** None.

## Additional file

Additional file 1: Supplementary Material (including supplementary figures and tables) for PaSS. (DOCX 4780 kb)
