# Supplementary material for: PaSS: a sequencing simulator for PacBio sequencing
Source: BMC Bioinformatics. 2019 Jun 21;20:352. doi: 10.1186/s12859-019-2901-7 (PMC6588853; doi:10.1186/s12859-019-2901-7)
Supplement: Supplementary file 1 — Supplementary Material (including supplementary figures and tables) for PaSS. (DOCX 4780 kb) [file 12859_2019_2901_MOESM1_ESM.docx]

**Additional file for**

**PaSS: A sequencing simulator for PacBio sequencing**

Wenmin Zhang, Ben Jia, Chaochun Wei

There are 8 supplementary tables and 9 supplementary figures included in this file.

Table S1. Sequencing codon bias or context-specific bins derived from real sequencing datasets *E. coli K12*, *C. elegans* and *A. thaliana.*

Table S2. The comparison of different preset error rates for unaligned part.

Table S3. Information about the three real PacBio sequencing datasets.

Table S4. Comparison of read length distributions between simulated reads and the real sequencing data.

Table S5. Comparison of error size distributions between simulated reads and the real sequencing data.

Table S6. Comparison of simulated reads to the real sequencing data for *C. elegans*.

Table S7. Comparison of simulated reads to the real sequencing data for *A.thaliana*.

Table S8. Comparison of speed for different simulation programs.

Figure S1. Ratio distribution of unaligned part in head and tail regions.

Figure S2.The ratio of insertion(a),deletion(b) and substitution(c) for 64 kinds of k-mers in real datasets *E.coli K12, C.elegans* and *A.thaliana*.

Figure S3. Comparison of error size distributions for different error types.

Figure S4. The cumulative distribution of read length for datasets *E.coli K12*, *C.elegans* and *A.thaliana*

Figure S5. The comparison of accumulative distributions of error sizes.

Figure S6. Comparison of the distributions of error rates along relative positions of a read.

Figure S7. Distribution of the average accuracy over the whole read for datasets *E.coli K12*, *C.elegans* and *A.thaliana.*

Figure S8. Comparison of the assembly results for real sequencing reads and simulated reads by different methods for *C. elegans*.

Figure S9. Comparison of the assembly results for real sequencing reads and simulated reads by different methods for *A.thaliana*.

Table S1. Sequencing codon bias or context-specific bins derived from real sequencing datasets *E. coli K12*, *C. elegans* and *A. thaliana*. It is presented in ascending order of the ratio of matches in each dataset.

| kmer | *E. coli K12* | | | |  | kmer | *C. elegans* | | | |
| --- | --- | --- | --- | --- | --- | --- | --- | --- | --- | --- |
|  | match | insertion | deletion | substitution |  |  | match | insertion | deletion | substitution |
| CCC | 0.713 | 0.080 | 0.177 | 0.030 |  | CCC | 0.721 | 0.064 | 0.186 | 0.029 |
| CCT | 0.779 | 0.075 | 0.119 | 0.027 |  | GGG | 0.789 | 0.048 | 0.147 | 0.016 |
| CCG | 0.784 | 0.081 | 0.108 | 0.028 |  | CCT | 0.794 | 0.060 | 0.123 | 0.024 |
| CCA | 0.784 | 0.073 | 0.117 | 0.026 |  | CCG | 0.804 | 0.067 | 0.106 | 0.024 |
| GGG | 0.814 | 0.066 | 0.109 | 0.011 |  | CCA | 0.804 | 0.061 | 0.112 | 0.023 |
| AAA | 0.814 | 0.079 | 0.093 | 0.014 |  | AAA | 0.837 | 0.065 | 0.085 | 0.013 |
| TTT | 0.824 | 0.122 | 0.048 | 0.006 |  | GGC | 0.839 | 0.050 | 0.098 | 0.013 |
| AAC | 0.854 | 0.073 | 0.059 | 0.013 |  | TTT | 0.840 | 0.079 | 0.072 | 0.010 |
| AAT | 0.860 | 0.069 | 0.058 | 0.013 |  | GGT | 0.845 | 0.044 | 0.098 | 0.013 |
| GGC | 0.865 | 0.063 | 0.063 | 0.008 |  | GGA | 0.850 | 0.042 | 0.096 | 0.013 |
| GGT | 0.866 | 0.057 | 0.069 | 0.009 |  | AAC | 0.879 | 0.060 | 0.049 | 0.012 |
| GGA | 0.875 | 0.052 | 0.064 | 0.008 |  | AAG | 0.884 | 0.058 | 0.046 | 0.012 |
| AAG | 0.876 | 0.066 | 0.045 | 0.013 |  | CTC | 0.890 | 0.056 | 0.038 | 0.016 |
| CTC | 0.879 | 0.070 | 0.034 | 0.017 |  | AAT | 0.892 | 0.052 | 0.043 | 0.012 |
| CAC | 0.879 | 0.075 | 0.031 | 0.015 |  | CAC | 0.894 | 0.057 | 0.035 | 0.014 |
| CGC | 0.881 | 0.071 | 0.029 | 0.018 |  | CGC | 0.896 | 0.057 | 0.031 | 0.016 |
| TTG | 0.882 | 0.087 | 0.027 | 0.005 |  | CTG | 0.898 | 0.054 | 0.033 | 0.014 |
| TTC | 0.882 | 0.084 | 0.029 | 0.005 |  | TTG | 0.898 | 0.052 | 0.041 | 0.008 |
| CTT | 0.885 | 0.062 | 0.031 | 0.022 |  | CTT | 0.899 | 0.047 | 0.037 | 0.018 |
| CTA | 0.888 | 0.062 | 0.034 | 0.016 |  | CTA | 0.900 | 0.051 | 0.034 | 0.015 |
| TTA | 0.889 | 0.079 | 0.028 | 0.005 |  | TTC | 0.901 | 0.052 | 0.040 | 0.008 |
| CGA | 0.889 | 0.065 | 0.029 | 0.017 |  | CGT | 0.902 | 0.051 | 0.031 | 0.016 |
| CTG | 0.889 | 0.068 | 0.027 | 0.016 |  | CGA | 0.903 | 0.052 | 0.030 | 0.015 |
| CGT | 0.890 | 0.064 | 0.028 | 0.017 |  | TTA | 0.904 | 0.047 | 0.041 | 0.008 |
| CAT | 0.890 | 0.068 | 0.027 | 0.015 |  | CAT | 0.904 | 0.051 | 0.031 | 0.013 |
| CAG | 0.892 | 0.071 | 0.023 | 0.014 |  | CAG | 0.905 | 0.054 | 0.029 | 0.012 |
| ATC | 0.893 | 0.080 | 0.017 | 0.011 |  | CGG | 0.907 | 0.050 | 0.029 | 0.014 |
| CGG | 0.894 | 0.064 | 0.026 | 0.016 |  | CAA | 0.908 | 0.048 | 0.031 | 0.013 |
| CAA | 0.894 | 0.061 | 0.030 | 0.014 |  | ACA | 0.908 | 0.063 | 0.019 | 0.009 |
| ATG | 0.898 | 0.078 | 0.013 | 0.010 |  | ACG | 0.909 | 0.067 | 0.015 | 0.009 |
| ACC | 0.901 | 0.072 | 0.018 | 0.010 |  | GCA | 0.910 | 0.057 | 0.024 | 0.009 |
| ACG | 0.903 | 0.073 | 0.014 | 0.011 |  | ACC | 0.911 | 0.062 | 0.018 | 0.009 |
| ACA | 0.904 | 0.068 | 0.017 | 0.011 |  | GCG | 0.911 | 0.059 | 0.022 | 0.009 |
| ATT | 0.905 | 0.066 | 0.016 | 0.013 |  | ATG | 0.914 | 0.063 | 0.014 | 0.010 |
| ATA | 0.906 | 0.071 | 0.013 | 0.010 |  | GCC | 0.914 | 0.050 | 0.027 | 0.009 |
| AGC | 0.907 | 0.064 | 0.018 | 0.011 |  | ATA | 0.915 | 0.061 | 0.014 | 0.010 |
| ACT | 0.909 | 0.064 | 0.017 | 0.010 |  | ATC | 0.916 | 0.058 | 0.016 | 0.010 |
| AGG | 0.914 | 0.060 | 0.016 | 0.010 |  | AGC | 0.917 | 0.057 | 0.016 | 0.010 |
| AGA | 0.914 | 0.062 | 0.015 | 0.009 |  | GTG | 0.918 | 0.044 | 0.029 | 0.009 |
| GCG | 0.914 | 0.071 | 0.010 | 0.005 |  | GCT | 0.918 | 0.045 | 0.027 | 0.010 |
| AGT | 0.915 | 0.059 | 0.016 | 0.010 |  | AGA | 0.919 | 0.056 | 0.016 | 0.009 |
| GCA | 0.916 | 0.069 | 0.010 | 0.006 |  | GTT | 0.919 | 0.036 | 0.030 | 0.014 |
| GCC | 0.916 | 0.067 | 0.012 | 0.005 |  | ACT | 0.920 | 0.055 | 0.016 | 0.009 |
| GCT | 0.925 | 0.059 | 0.011 | 0.006 |  | GTC | 0.920 | 0.040 | 0.030 | 0.009 |
| TCG | 0.929 | 0.057 | 0.009 | 0.005 |  | AGG | 0.922 | 0.052 | 0.016 | 0.009 |
| TCT | 0.930 | 0.055 | 0.010 | 0.005 |  | ATT | 0.923 | 0.048 | 0.014 | 0.015 |
| GAC | 0.932 | 0.052 | 0.010 | 0.005 |  | GTA | 0.924 | 0.039 | 0.028 | 0.009 |
| GTT | 0.932 | 0.045 | 0.011 | 0.011 |  | AGT | 0.926 | 0.050 | 0.015 | 0.009 |
| TCC | 0.933 | 0.053 | 0.008 | 0.006 |  | GAC | 0.926 | 0.039 | 0.024 | 0.010 |
| GTC | 0.933 | 0.050 | 0.011 | 0.006 |  | GAG | 0.929 | 0.038 | 0.024 | 0.010 |
| TGC | 0.934 | 0.051 | 0.010 | 0.005 |  | GAT | 0.931 | 0.035 | 0.023 | 0.010 |
| GTG | 0.935 | 0.050 | 0.009 | 0.006 |  | TAC | 0.934 | 0.039 | 0.018 | 0.010 |
| TGT | 0.935 | 0.050 | 0.010 | 0.005 |  | TAT | 0.934 | 0.038 | 0.019 | 0.010 |
| TCA | 0.936 | 0.051 | 0.008 | 0.005 |  | GAA | 0.934 | 0.033 | 0.022 | 0.011 |
| TGG | 0.937 | 0.048 | 0.011 | 0.004 |  | TCT | 0.935 | 0.040 | 0.017 | 0.008 |
| TAC | 0.937 | 0.049 | 0.008 | 0.006 |  | TGT | 0.936 | 0.038 | 0.019 | 0.007 |
| GAG | 0.937 | 0.047 | 0.010 | 0.006 |  | TCG | 0.937 | 0.040 | 0.015 | 0.008 |
| TAT | 0.938 | 0.047 | 0.009 | 0.006 |  | TGC | 0.937 | 0.037 | 0.017 | 0.008 |
| GAT | 0.939 | 0.046 | 0.009 | 0.007 |  | TCC | 0.939 | 0.038 | 0.015 | 0.009 |
| TGA | 0.939 | 0.047 | 0.010 | 0.004 |  | TAG | 0.940 | 0.035 | 0.017 | 0.008 |
| GTA | 0.941 | 0.043 | 0.010 | 0.006 |  | TAA | 0.941 | 0.031 | 0.017 | 0.011 |
| TAG | 0.943 | 0.044 | 0.009 | 0.005 |  | TCA | 0.942 | 0.036 | 0.014 | 0.008 |
| GAA | 0.943 | 0.041 | 0.009 | 0.007 |  | TGG | 0.944 | 0.033 | 0.016 | 0.007 |
| TAA | 0.944 | 0.042 | 0.008 | 0.005 |  | TGA | 0.944 | 0.033 | 0.016 | 0.007 |

| kmer | *A. thaliana* | | | |
| --- | --- | --- | --- | --- |
|  | match | insertion | deletion | substitution |
| TTT | 0.798 | 0.103 | 0.039 | 0.060 |
| TTC | 0.818 | 0.082 | 0.027 | 0.073 |
| GGG | 0.820 | 0.084 | 0.078 | 0.018 |
| CCC | 0.820 | 0.100 | 0.050 | 0.029 |
| AAA | 0.822 | 0.075 | 0.083 | 0.021 |
| TTG | 0.827 | 0.073 | 0.028 | 0.072 |
| TTA | 0.830 | 0.068 | 0.030 | 0.072 |
| TCC | 0.843 | 0.063 | 0.014 | 0.080 |
| GGC | 0.843 | 0.083 | 0.055 | 0.019 |
| TAC | 0.844 | 0.053 | 0.016 | 0.087 |
| TGC | 0.844 | 0.056 | 0.017 | 0.083 |
| TCT | 0.847 | 0.060 | 0.011 | 0.082 |
| TCG | 0.850 | 0.056 | 0.013 | 0.081 |
| TAT | 0.851 | 0.049 | 0.017 | 0.083 |
| CCT | 0.853 | 0.082 | 0.034 | 0.030 |
| TCA | 0.854 | 0.051 | 0.013 | 0.082 |
| CCG | 0.854 | 0.087 | 0.029 | 0.030 |
| TAA | 0.854 | 0.044 | 0.022 | 0.079 |
| TGT | 0.855 | 0.055 | 0.017 | 0.074 |
| GGT | 0.857 | 0.071 | 0.055 | 0.017 |
| CCA | 0.858 | 0.077 | 0.035 | 0.030 |
| AAC | 0.859 | 0.073 | 0.050 | 0.018 |
| TAG | 0.860 | 0.044 | 0.016 | 0.080 |
| TGG | 0.861 | 0.047 | 0.020 | 0.072 |
| TGA | 0.861 | 0.043 | 0.020 | 0.076 |
| AAT | 0.861 | 0.061 | 0.058 | 0.020 |
| GGA | 0.862 | 0.063 | 0.057 | 0.018 |
| GCA | 0.865 | 0.098 | 0.022 | 0.015 |
| AAG | 0.865 | 0.062 | 0.054 | 0.019 |
| GCC | 0.872 | 0.088 | 0.022 | 0.018 |
| GCG | 0.874 | 0.091 | 0.018 | 0.017 |
| CGC | 0.880 | 0.079 | 0.009 | 0.032 |
| ACC | 0.883 | 0.085 | 0.016 | 0.015 |
| GCT | 0.886 | 0.073 | 0.023 | 0.018 |
| CTC | 0.889 | 0.075 | 0.009 | 0.027 |
| CAC | 0.889 | 0.072 | 0.011 | 0.027 |
| ATG | 0.889 | 0.070 | 0.023 | 0.018 |
| ACG | 0.890 | 0.077 | 0.018 | 0.015 |
| CGG | 0.890 | 0.070 | 0.010 | 0.031 |
| ATC | 0.892 | 0.068 | 0.022 | 0.018 |
| ATA | 0.893 | 0.067 | 0.022 | 0.018 |
| GTT | 0.893 | 0.063 | 0.023 | 0.020 |
| CTT | 0.894 | 0.063 | 0.011 | 0.031 |
| ACA | 0.894 | 0.073 | 0.018 | 0.015 |
| ATT | 0.895 | 0.059 | 0.023 | 0.023 |
| CGA | 0.896 | 0.068 | 0.009 | 0.028 |
| ACT | 0.896 | 0.068 | 0.020 | 0.017 |
| CGT | 0.898 | 0.065 | 0.008 | 0.028 |
| CTA | 0.899 | 0.062 | 0.010 | 0.029 |
| CTG | 0.899 | 0.065 | 0.009 | 0.027 |
| CAA | 0.899 | 0.062 | 0.013 | 0.025 |
| CAG | 0.900 | 0.065 | 0.011 | 0.025 |
| AGC | 0.900 | 0.060 | 0.020 | 0.020 |
| CAT | 0.900 | 0.062 | 0.011 | 0.027 |
| GTC | 0.900 | 0.057 | 0.025 | 0.018 |
| GAC | 0.902 | 0.054 | 0.024 | 0.020 |
| GTG | 0.903 | 0.058 | 0.023 | 0.016 |
| GTA | 0.904 | 0.053 | 0.026 | 0.017 |
| GAG | 0.906 | 0.052 | 0.025 | 0.018 |
| AGT | 0.907 | 0.052 | 0.021 | 0.019 |
| GAT | 0.907 | 0.050 | 0.024 | 0.018 |
| GAA | 0.908 | 0.045 | 0.027 | 0.020 |
| AGG | 0.908 | 0.053 | 0.020 | 0.019 |
| AGA | 0.908 | 0.057 | 0.018 | 0.016 |

Table S2. The comparison of different preset error rates for unaligned part. We selected 0.4 as the present error rate. In order to determine the error rate of the unaligned regions, a fixed error rate was applied for all bases in each read. For each error rate, 50,000 reads were simulated for genome *E. coli* *K12*. The reads were then aligned to *E. coli K12* genome and calculated the proportion of the reads and bases that can be aligned. No base should be aligned back to the reference genome if the error rate is higher than the error rate that we will adopt as the preset error rate for unaligned regions. We tried error rates of 0.3, 04, 0.5 and 0.6, and selected 0.4 as the default preset error rate since the percentage of aligned bases reduced dramatically to less than 1% after the error rate was set to 0.4.

| preset error rate | mapped_reads % | aligned_bases% |
| --- | --- | --- |
| 0.3 | 80.52% | 83.10% |
| 0.4 | 23.11% | 0.80% |
| 0.5 | 11.70% | 0.16% |
| 0.6 | 0.57% | 0.01% |

Table S3. Information about the three real PacBio sequencing datasets.

| datasets | platform | #run | #reads | #bases | read length (bases) | | | | URL |
| --- | --- | --- | --- | --- | --- | --- | --- | --- | --- |
|  |  |  |  |  | min | max | average | sigma |  |
| *E. coli K12* | RSII P6-C4 | 1 | 87,217 | 750M | 1 | 44,113 | 8,582 | 6,953.4 | a |
| *C. elegans* | RSII P6-C4 | 11 | 420,745 | 4.6G | 35 | 54,979 | 11,241 | 7,640.1 | b |
| *A. thaliana* | Sequel | 1 | 561,176 | 5.5G | 50 | 70,763 | 9,633 | 7,923.4 | c |

a. https://github.com/PacificBiosciences/DevNet/wiki/E.-coli-Bacterial-Assembly

b. <https://github.com/PacificBiosciences/DevNet/wiki/C.-elegans-data-set>

c. https://downloads.pacbcloud.com/public/SequelData/ArabidopsisDemoData/

Table S4. Comparison of read length distributions between simulated reads and the real sequencing data. Kolmogorov–Smirnov test was applied. Simulated data of different simulators and real sequencing data were compared based on their read length distributions.

| Datasets | Methods | Test statistics D | p-value |
| --- | --- | --- | --- |
| *E. coli K12* | PBSIM | 0.0225 | <2.2e-16 |
|  | LongISLND | 0.0235 | <2.2e-16 |
|  | NPBSS | 0.1282 | <2.2e-16 |
|  | PaSS | 0.0100 | 0.0003 |
| *C. elegans* | PBSIM | 0.0416 | <2.2e-16 |
|  | LongISLND | 0.0114 | <2.2e-16 |
|  | NPBSS | 0.2543 | <2.2e-16 |
|  | PaSS | 0.0095 | <2.2e-16 |
| *A. thaliana* | PBSIM | 0.0505 | <2.2e-16 |
|  | NPBSS | 0.1439 | <2.2e-16 |
|  | PaSS | 0.0233 | <2.2e-16 |

Table S5 Comparison of error size distributions between simulated reads and the real sequencing data. Kolmogorov–Smirnov test was applied to compare simulated data of different simulators and real sequencing data on error size distributions.

| Datasets | Methods | Insertion length | | Deletion length | | Substitution length | |
| --- | --- | --- | --- | --- | --- | --- | --- |
|  |  | Test  statistics D | p-value | Test  statistics D | p-value | Test  statistics D | p-value |
| *E. coli K12* | PBSIM | 0.0642 | <2.2e-16 | 0.0556 | <2.2e-16 | 0.0158 | <2.2e-16 |
|  | LongISLND | 0.0295 | <2.2e-16 | 0.0363 | <2.2e-16 | 0.0197 | <2.2e-16 |
|  | NPBSS | 0.1521 | <2.2e-16 | 0.0051 | <2.2e-16 | 0.0103 | <2.2e-16 |
|  | PaSS | 0.0035 | <2.2e-16 | 0.0293 | <2.2e-16 | 0.0054 | <2.2e-16 |
| *C. elegans* | PBSIM | 0.0505 | <2.2e-16 | 0.0750 | <2.2e-16 | 0.0165 | <2.2e-16 |
|  | LongISLND | 0.0376 | <2.2e-16 | 0.0357 | <2.2e-16 | 0.0198 | <2.2e-16 |
|  | NPBSS | 0.1339 | <2.2e-16 | 0.0120 | <2.2e-16 | 0.0090 | <2.2e-16 |
|  | PaSS | 0.0019 | <2.2e-16 | 0.0373 | <2.2e-16 | 0.0050 | <2.2e-16 |
| *A. thaliana* | PBSIM | 0.1408 | <2.2e-16 | 0.0296 | <2.2e-16 | 0.0014 | <2.2e-16 |
|  | NPBSS | 0.1826 | <2.2e-16 | 0.0143 | <2.2e-16 | 0.0363 | <2.2e-16 |
|  | PaSS | 0.0079 | <2.2e-16 | 0.0370 | <2.2e-16 | 0.0038 | <2.2e-16 |

Table S6. Comparison of simulated reads to the real sequencing data for *C. elegans*. Statistics about the simulated reads by PBSIM, LongISLND, PaSS and real sequencing data were calculated by aligning the reads back to *C. elegans* genome.

| methods | aligned  rate(read) | aligned  rate(base) | error  rate(%) | insertion(%) | deletion(%) | substitution(%) |
| --- | --- | --- | --- | --- | --- | --- |
| real data | 97.50% | 91.97% | 14.62% | 8.27% | 4.97% | 1.38% |
| PBSIM | 99.97% | 99.77% | 12.22% | 7.14% | 3.14% | 1.94% |
| LongISLND | 99.89% | 99.95% | 10.71% | 5.48% | 3.93% | 1.30% |
| NPBSS | 99.99% | 99.90% | 11.60% | 2.69% | 6.09% | 2.82% |
| PaSS | 96.83% | 93.82% | 13.44% | 7.30% | 4.50% | 1.62% |

Table S7. Comparison of simulated reads to the real sequencing data for *A.thaliana*. Statistics about the simulated reads by PBSIM, NPBSS, PaSS and real sequencing data were calculated by aligning the reads back to *A.thaliana* high-quality de-novo assembly contigs.

| methods | aligned  rate(read) | aligned  rate(base) | error  rate(%) | insertion(%) | deletion(%) | substitution(%) |
| --- | --- | --- | --- | --- | --- | --- |
| real data | 93.96% | 88.69% | 18.27% | 10.59% | 3.65% | 4.03% |
| PBSIM | 99.98% | 98.66% | 0.91% | 0.48% | 0.29% | 0.14% |
| NPBSS | 99.76% | 99.89% | 11.54% | 2.67% | 6.08% | 2.80% |
| PaSS | 96.06% | 90.81% | 17.27% | 10.02% | 3.66% | 3.60% |

Table S8. Comparison of speed for different simulation programs. Running time of PBSIM,LongISLND,NPBSS and PaSS to simulate *E. coli* K12 genome (left part) and *C. elegans* genome (right part) with sequencing depth 170 and 50 respectively.

| *E. coli* K12 |  |  | *C. elegans* |  |
| --- | --- | --- | --- | --- |
| Simulators | Time |  | Simulators | Time |
| PBSIM | 36s |  | PBSIM | 250s |
| LongISLND | 60s |  | LongISLND | 566s |
| NPBSS | 1380s |  | NPBSS | 7680s |
| PaSS_thread1 | 130s |  | PaSS_thread1 | 911s |
| PaSS_thread2 | 68s |  | PaSS_thread2 | 480s |
| PaSS_thread4 | 38s |  | PaSS_thread4 | 252s |
| PaSS_thread8 | 23s |  | PaSS_thread8 | 155s |
| PaSS_thread16 | 15s |  | PaSS_thread16 | 103s |


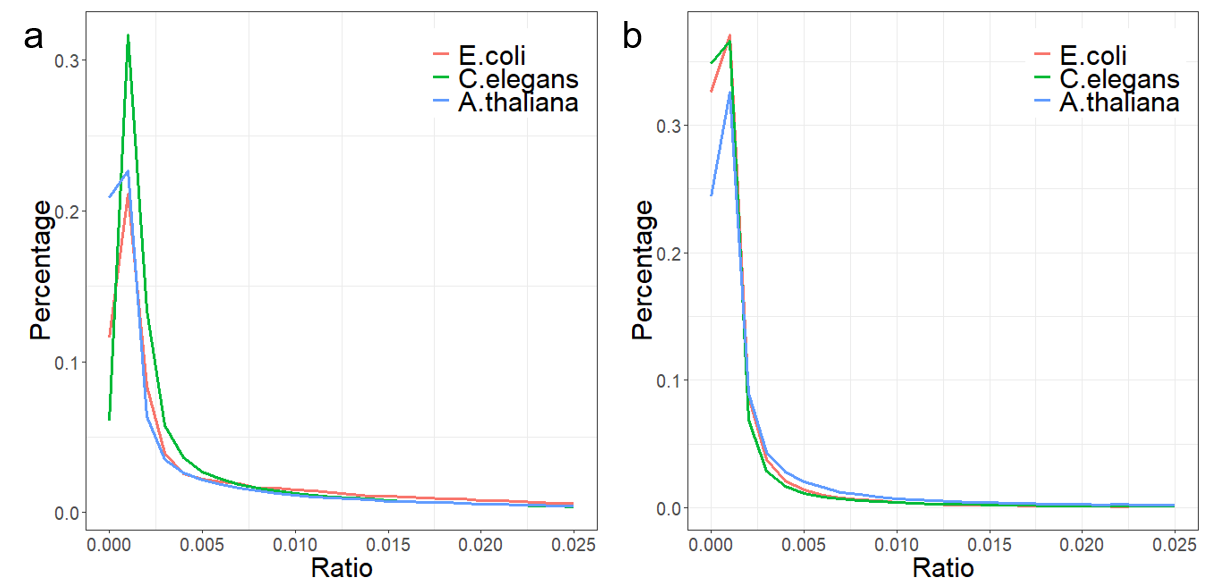


Fig. S1. Distribution of the ratio of unaligned parts（% of the total read length） in head and tail regions of three real datasets. X-axis stands for the ratio of the unaligned lengths in the head (a) and tail (b) regions of the polymerase reads

.
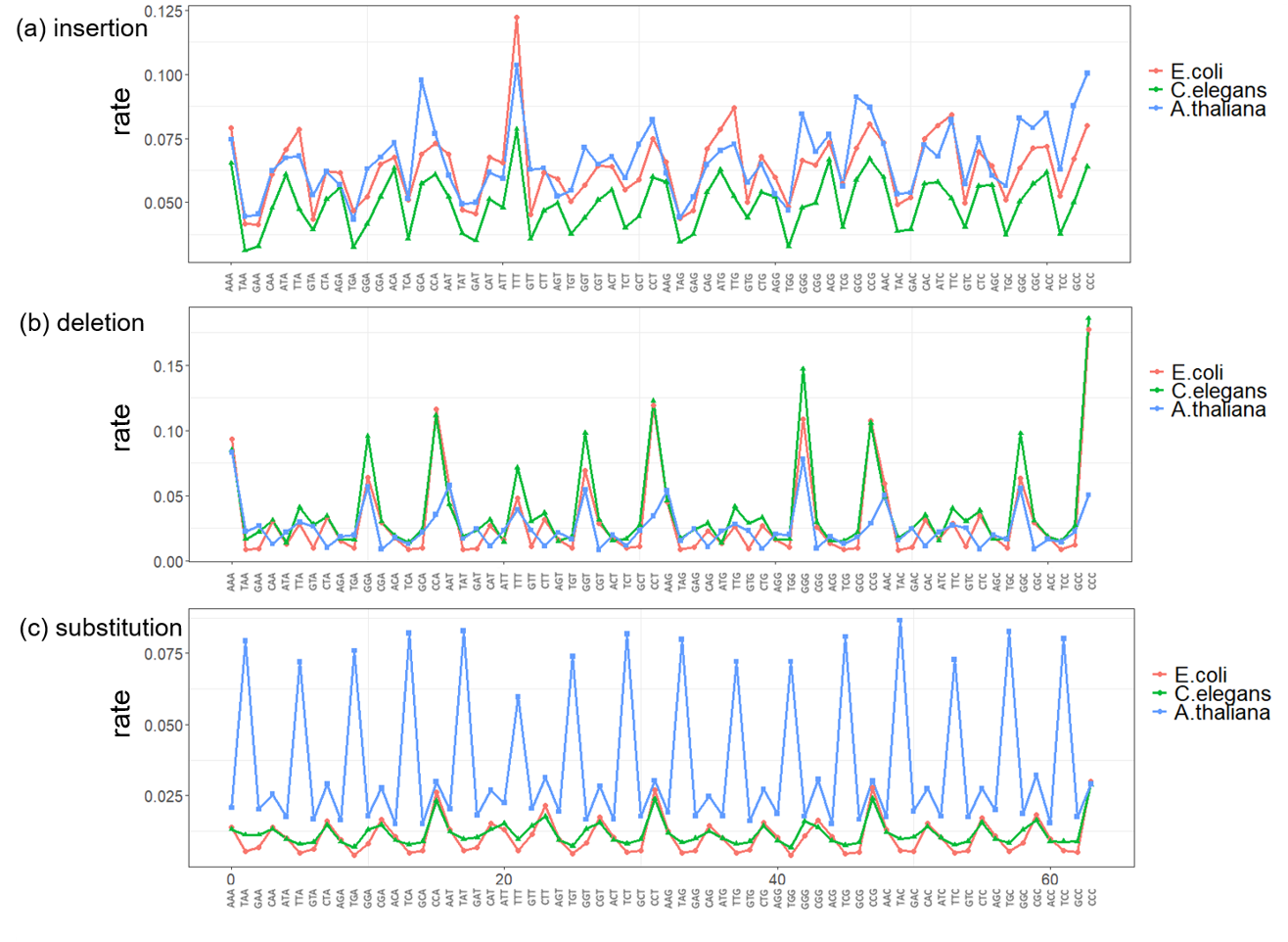


Fig. S2.The ratio of insertions (a), deletions (b) and substitutions (c) based on the context of 64 k-mers (k=3) in real datasets *E. coli* *K12*, *C. elegans* and *A. thaliana*.


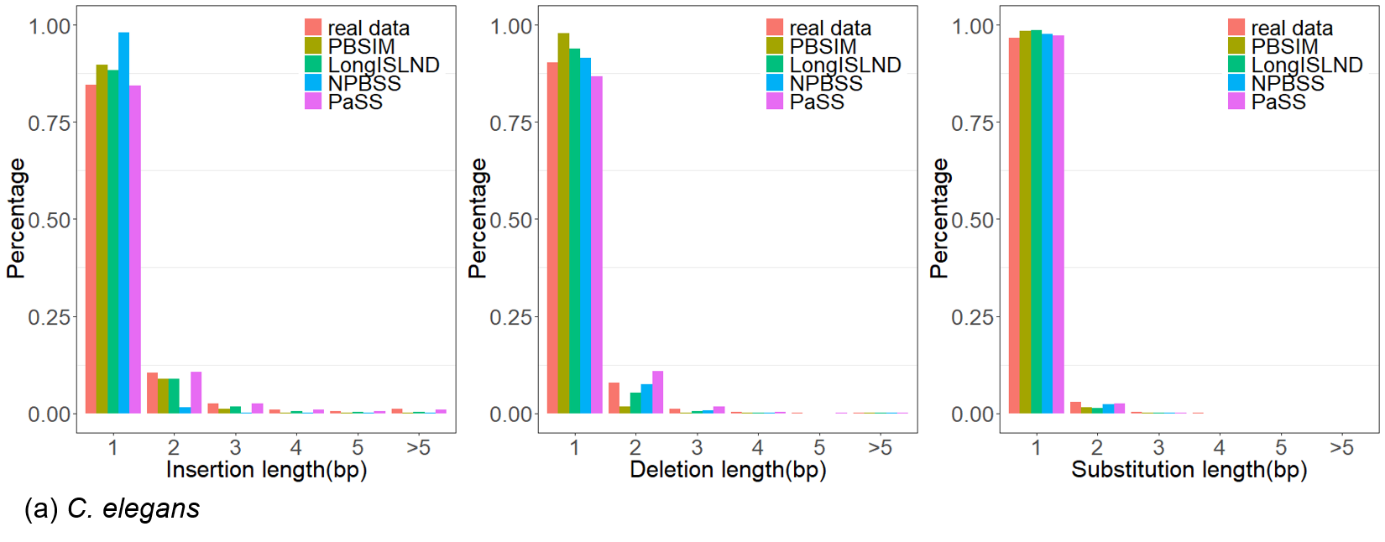


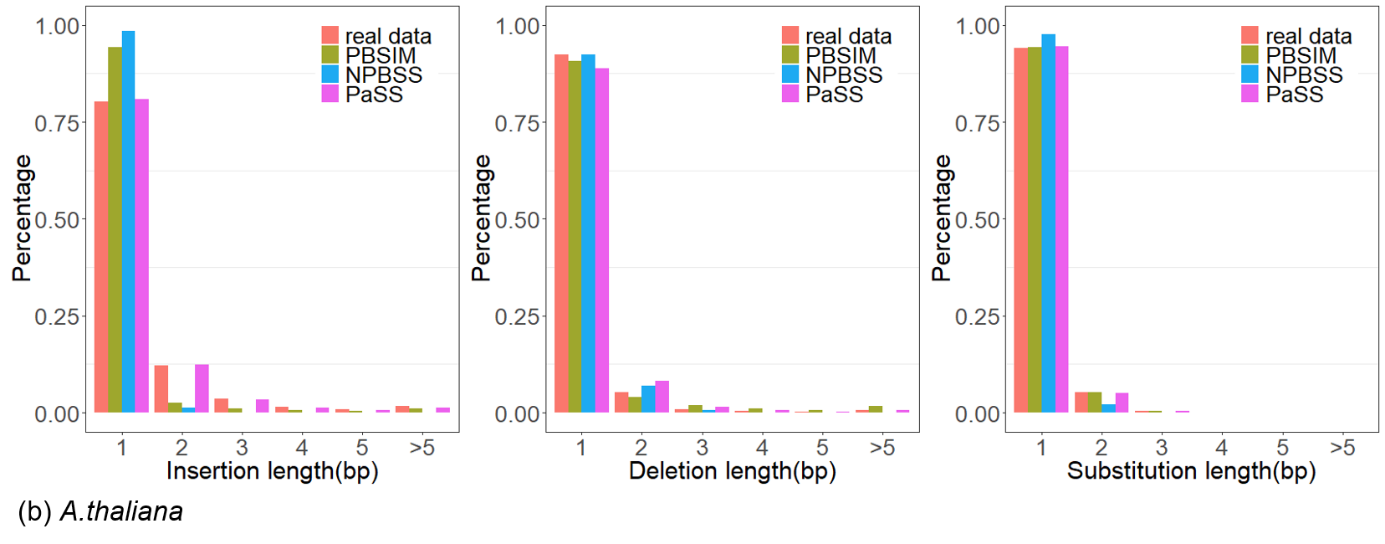


Fig. S3. Comparison of error size distributions for different error types. Simulated reads from different methods were compared with experimental data on error size distribution for dataset *C. elegans* and *A. thaliana*. Three subfigures showed the probability density bar plot for insertion, deletion and substitution respectively.


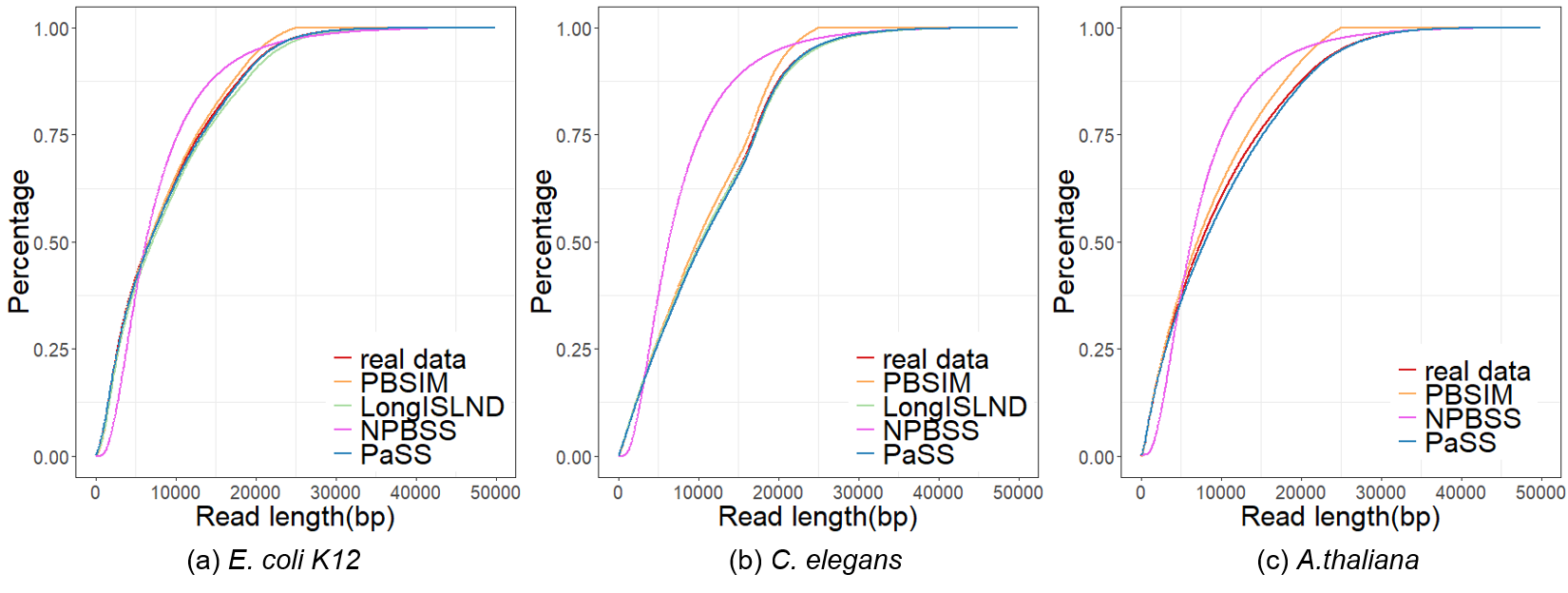


Fig. S4 The cumulative distribution plots of read length in datasets *E.coli K12*, *C.elegans* and *A.thaliana .*


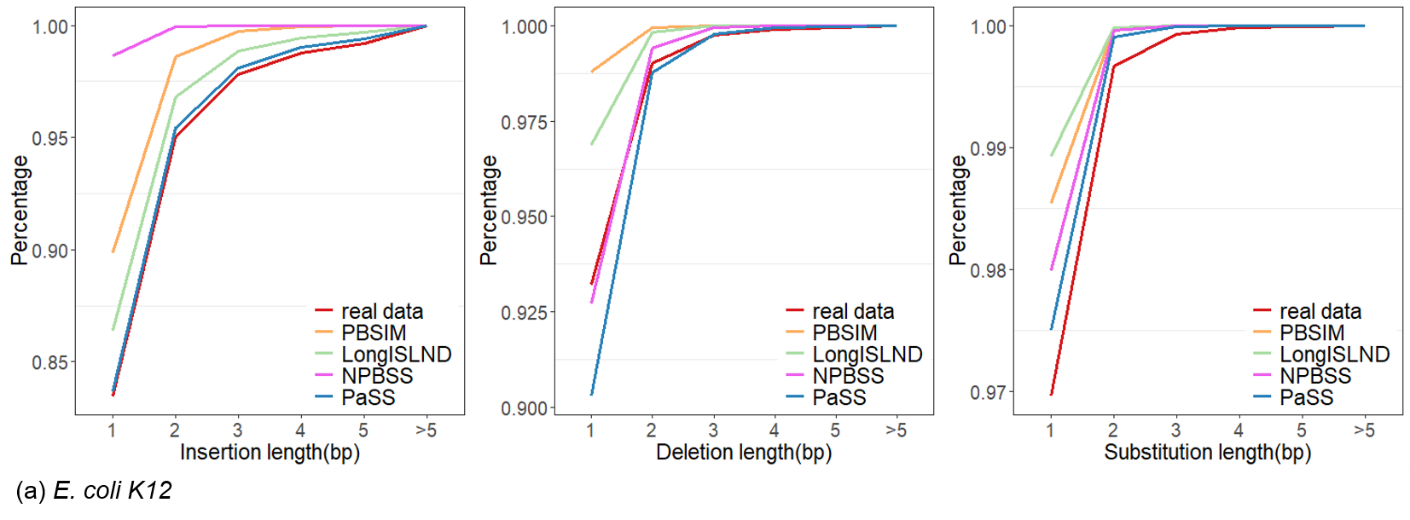


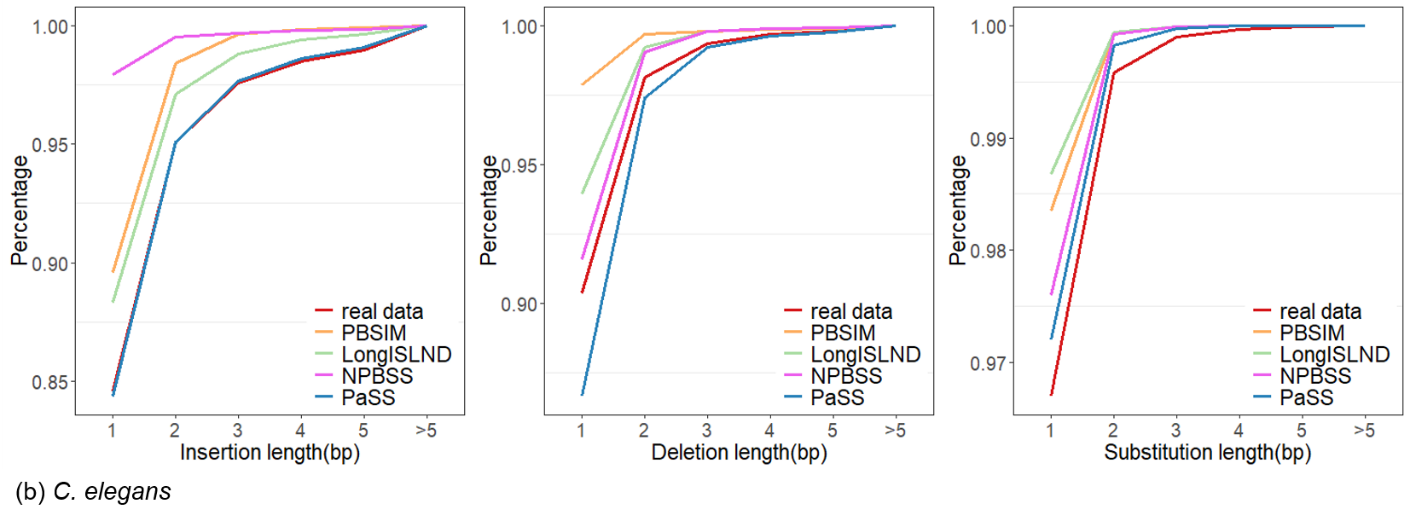


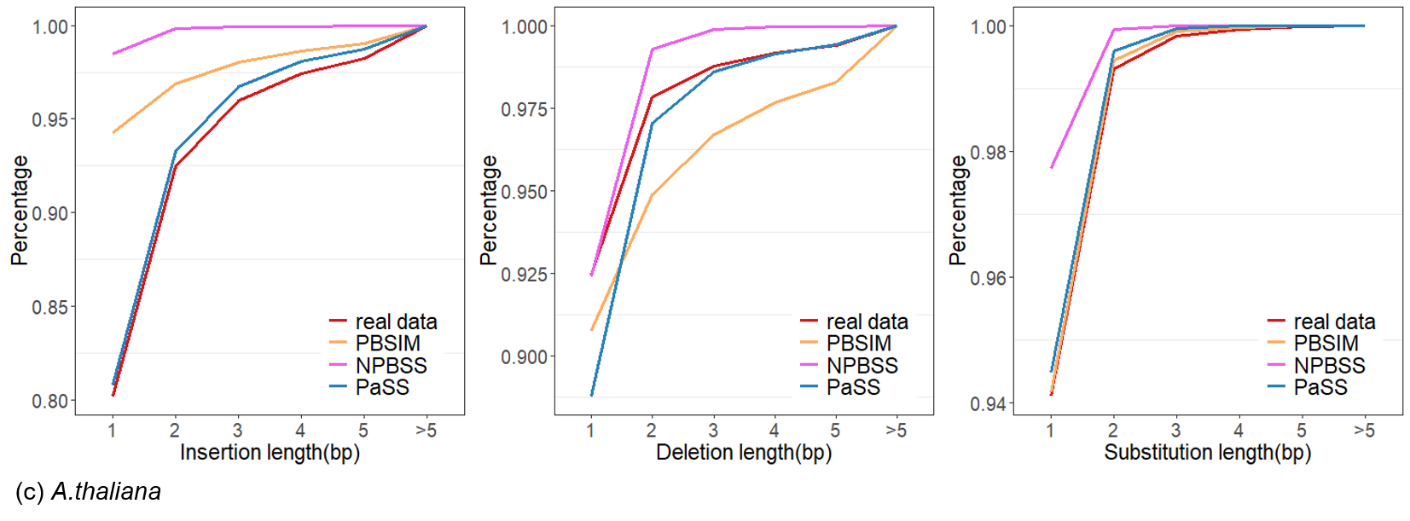


Fig. S5 The comparison of accumulative distributions of error sizes. The cumulative distribution plots of error size distributions in datasets *E.coli K12* (a), *C.elegans*(b) and *A.thaliana* (c). The distributions of lenghts of insertion, deletion and substitution were shown for each dataset.


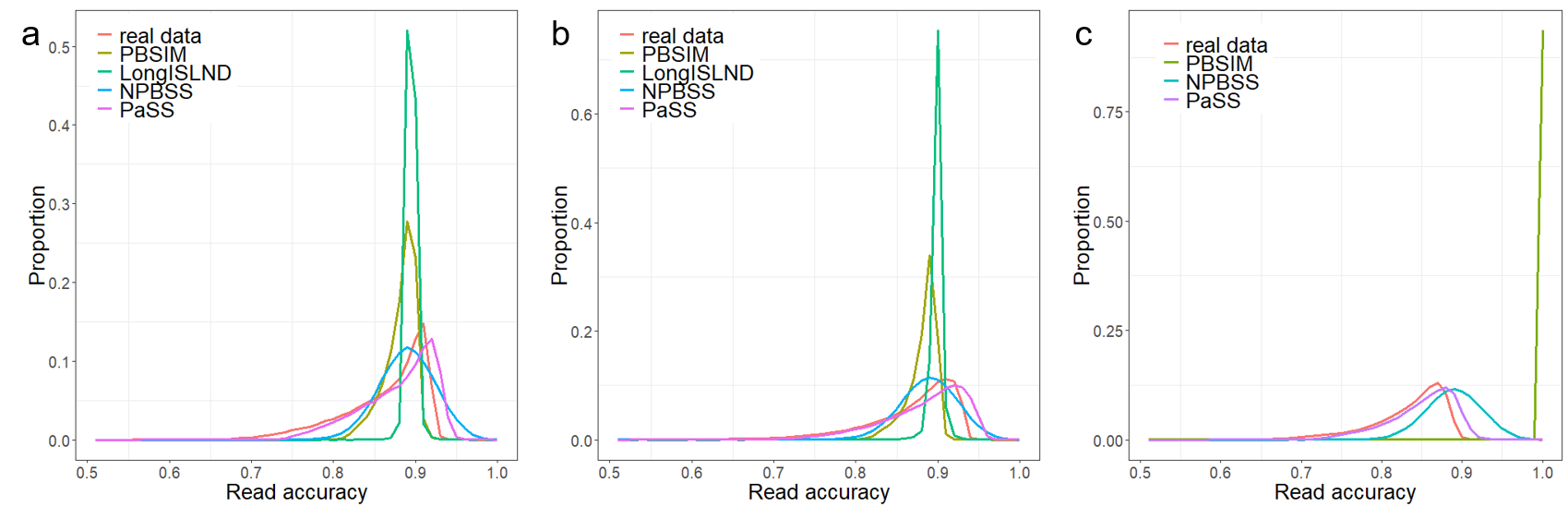


Fig. S6. Distribution of the average accuracy (1- error rate) over the whole read in datasets *E.coli K12*, *C.elegans* and *A.thaliana*


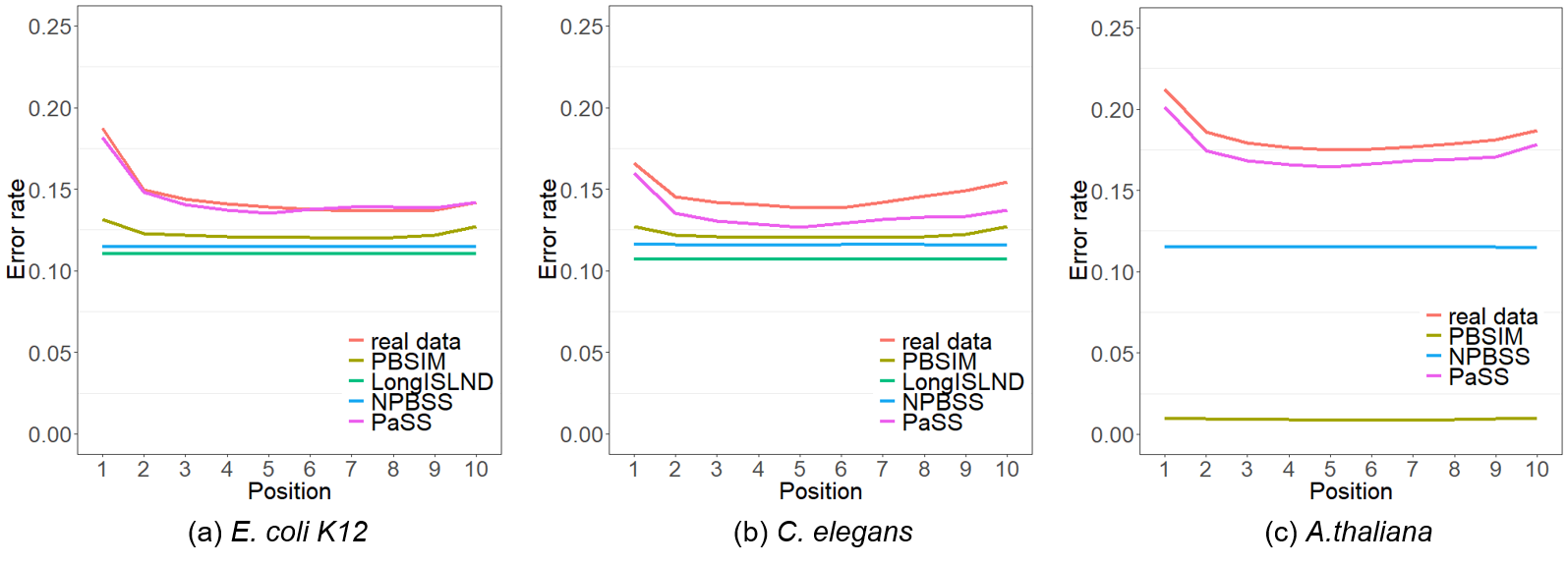


Figure S7. Comparison of the distributions of error rates along relative positions of a read. Each curve showed the relationship of the error rate and the relative position of a polymerase read in a dataset. X-axis stands for the relative positions along a polymerase read, which was evenly divided into 10 intervals. Y-axis stands for the average error rate of the bases inside the corresponding segment. Results show that PaSS has the most similar error rate distribution as the real sequencing data.


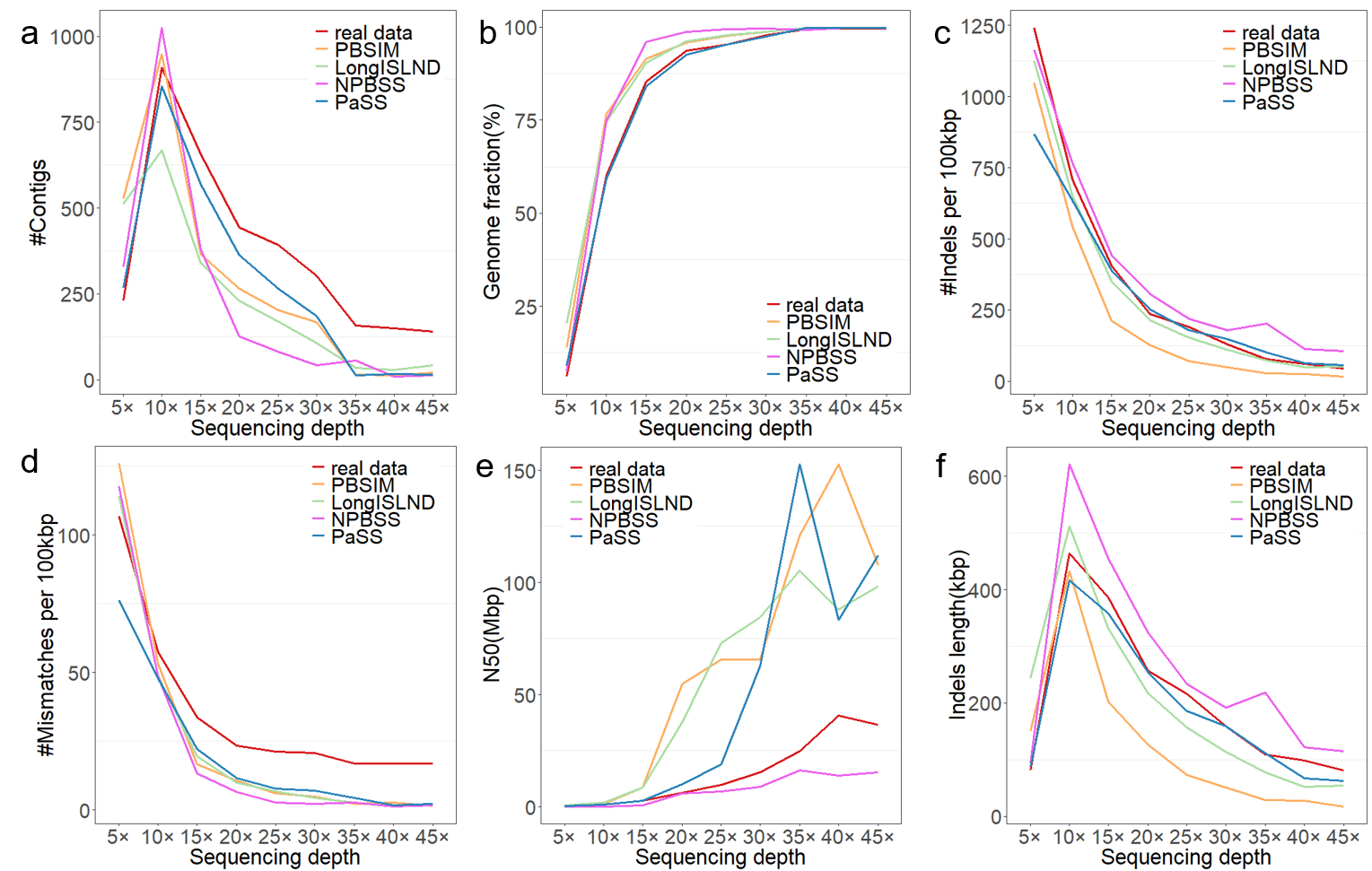


Fig. S8. Comparison of the assembly results for real reads and simulated reads by different methods for *C. elegans*. The six subfigures show the relationships between sequencing depth and the resulted (a) number of contigs, (b) genome fraction, (c) number of indels per 100kbp, (d) number of mismatches per 100kbp, (e) N50, and (f) the length of indels of contigs in the assembly results for *C. elegans* dataset. X-axis stands for the sequencing depth.


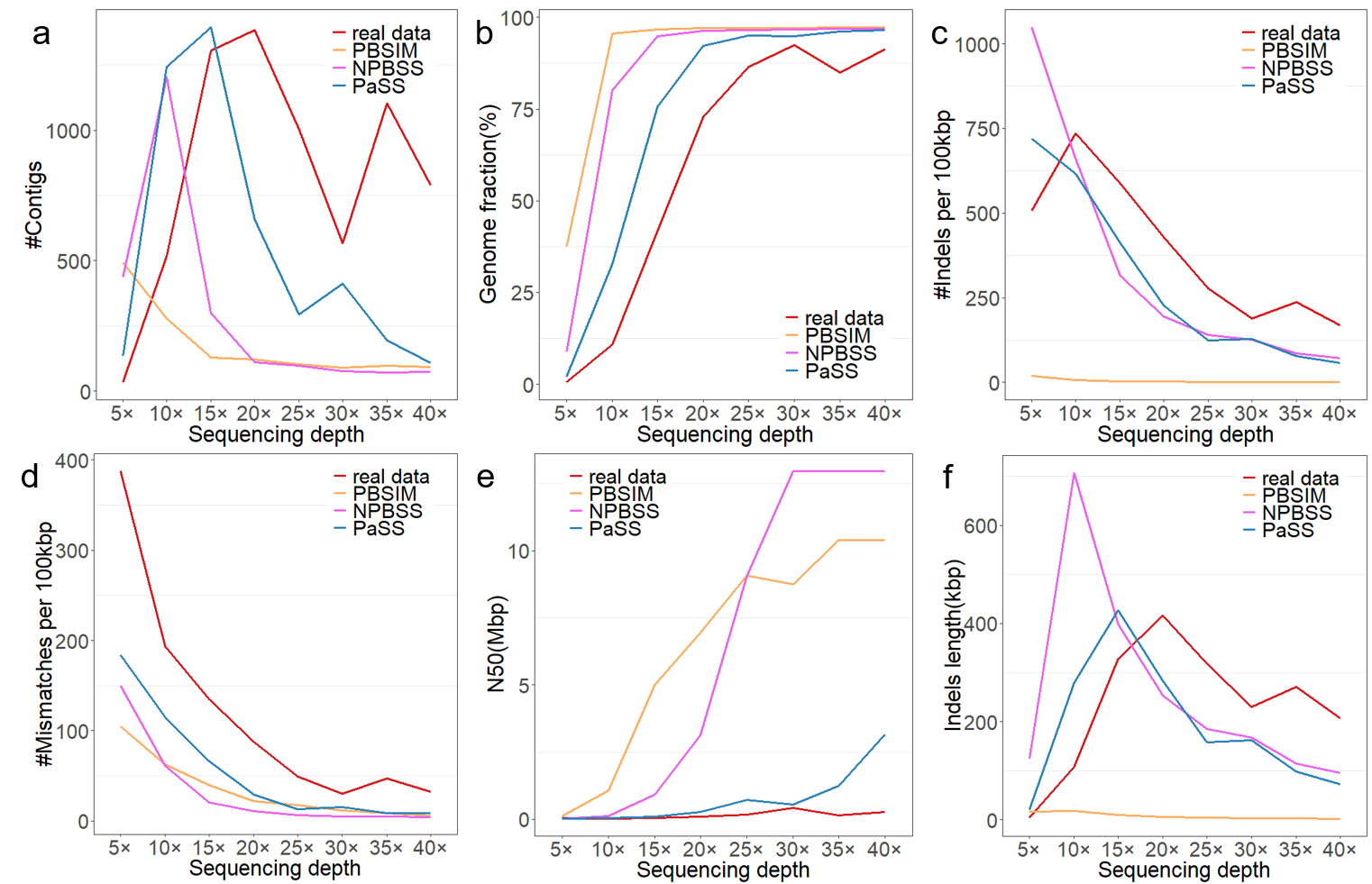


Fig. S9. Comparison of the assembly results for real sequencing reads and simulated reads by different methods for *A.thaliana*. The six subfigures show the relationships between sequencing depth and the resulted (a) number of contigs, (b) genome fraction, (c) number of indels per 100kbp, (d) number of mismatches per 100kbp, (e) N50, and (f) the length of indels of contigs in the assembly results for *A.thaliana* dataset. X-axis stands for the sequencing depth.
